# Electrochemical Conversion of CO_2_ to CO Utilizing Quaternized Polybenzimidazole Anion Exchange Membrane

**DOI:** 10.3390/membranes13020166

**Published:** 2023-01-29

**Authors:** Jingfeng Li, Zeyu Cao, Bo Zhang, Xinai Zhang, Jinchao Li, Yaping Zhang, Hao Duan

**Affiliations:** 1 State Key Laboratory of Environment-Friendly Energy Materials, Engineering Research Center of Biomass Materials (Ministry of Education), School of Materials Chemistry, Southwest University of Science and Technology, Mianyang 621010, China; 2 School of Chemical Engineering, Sichuan University, Chengdu 610065, China; 3 Sichuan Langsheng New Energy Technology Co., Ltd., Suining 629200, China

**Keywords:** membrane, quaternized polybenzimidazole, electrocatalytic reduction, carbon dioxide

## Abstract

CO is a significant product of electrochemical CO_2_ reduction (ECR) which can be mixed with H_2_ to synthesize numerous hydrocarbons. Membranes, as separators, can significantly influence the performance of ECR. Herein, a series of quaternized polybenzimidazole (QAPBI) anion exchange membranes with different quaternization degrees are prepared for application in ECR. Among all QAPBI membranes, the QAPBI-2 membrane exhibits optimized physico-chemical properties. In addition, the QAPBI-2 membrane shows higher a Faraday efficiency and CO partial current density compared with commercial Nafion 117 and FAA-3-PK-130 membranes, at −1.5 V (vs. RHE) in an H-type cell. Additionally, the QAPBI-2 membrane also has a higher Faraday efficiency and CO partial current density compared with Nafion 117 and FAA-3-PK-130 membranes, at −3.0 V in a membrane electrode assembly reactor. It is worth noting that the QAPBI-2 membrane also has excellent ECR stability, over 320 h in an H-type cell. This work illustrates a promising pathway to obtaining cost-effective membranes through a molecular structure regulation strategy for ECR application.

## 1. Introduction

Fossil fuels have been widely used to meet rapidly increasing energy demands with the advancement of society, leading to the emission of large amounts of CO_2_. This results in global warming and ocean acidification, which threaten the living environment of human beings. Thus, CO_2_ should be converted into high-added-value products through various methods [1]. Among all paths, electrochemical CO_2_ reduction (ECR) has intrigued scientific community because it can be accomplished at room temperature and atmospheric pressure, the reduction products can be adjusted, and the renewable energies can be stored in chemical products [2,3,4]. Among a variety of ECR products, CO, as an important component of syngas, could be converted into hydrocarbons through the Fisher–Topsch process [5,6,7]. Thus, the conversion of CO_2_ to CO has great significance for the practical application of ECR. In addition, the electrochemical conversion of CO_2_ to CO through a two-electron and two-proton reaction is relatively easy to accomplish [8,9]. The reactions and side reactions of the electrochemical reduction of CO_2_ to CO are described in Table 1.

The membrane is a core component of ECR devices which not only prevents the cross-contamination of anode and cathode electrolytes, but also transports specific ions to form a complete circuit [10]. Additionally, the membrane in an ECR has significant effects on the current density, Faraday efficiency and reduction product. Currently, the cation exchange membranes (such as Nafion series membranes) transfer protons from anode to cathode which then react with CO_2_ to produce CO [11]. Nevertheless, Nafion membranes have high potassium ion permeability and undergo a serious hydrogen evolution reaction which markedly reduces the Faraday efficiency of ECR [12]. Additionally, their high price also limits the large-scale commercial application of Nafion membranes in ECR. Fortunately, when anion exchange membranes such as quaternized polybenzimidazole (QAPBI) anion exchange membranes are applied in ECR, the hydrogen evolution reaction and potassium ion penetration can be effectively alleviated, since the transmission of H^+^ and K^+^ from anode to cathode is selectively restrained [13,14]. However, the reported QAPBI membranes show low conductivities, even with the dense hydrogen bond networks and/or the presence of cationic groups [15,16]. This result is mainly caused by an insufficient micro-phase separation structure, which suggests the extreme importance of the side chain design in a high-performance QAPBI anion exchange membrane. In addition, anion exchange membranes also display low stabilities, which inevitably becomes another major obstacle to their further utilization in ECR [17]. Disappointingly, there are only a few studies on membranes for ECR. Therefore, it is necessary to develop anion exchange membranes with reasonable cost, high OH^−^ conductivities and excellent durability for ECR application.

In this work, the QAPBI anion exchange membranes with a side-chain design are fabricated for application in ECR. Additionally, the quaternization degrees of QAPBI membranes are precisely controlled by adjusting the ratio of PBI and 2,3-epoxypropyltrimethylammonium chloride. To our knowledge, these QAPBI membranes with such novel molecular structures have not been reported in ECR. The QAPBI membranes are developed based on the following considerations: (i) the quaternary ammonium groups have strong OH^−^ conductivities compared to other cationic groups [18]; (ii) the C−O−C bonds and imidazole groups on the polybenzimidazole backbone can improve the OH^−^ conductivity [19]; and (iii) the polybenzimidazole backbone has high alkaline stability, owing to the substitution of N2 position on imidazole ring, which increases the steric hindrance and reduces the probability of OH^−^ attacking the C=N bond [20]. The QAPBI anion exchange membranes are systemically investigated, including their chemical structures, morphologies and physico-chemical properties. In addition, the ECR performances of PBI, optimal QAPBI and commercial anion/cation exchange membranes including the FAA-3-PK-130/Nafion 117 membrane are also thoroughly investigated.

## 2. Experimental

### 2.1. Reagents and Materials

Polybenzimidazole (Shanghai Shengjun Plastic Tech. Co., Ltd., Shanghai, China, 99.5%, AR), 2,3-epoxypropyltrimethylammonium chloride and silver foil (Shanghai Macklin Biochem. Tech. Co., Ltd., Shanghai, China, 95.0%, AR), dimethyl sulfoxide (Shanghai Rhawn Chem. Tech. Co., Ltd., Shanghai, China, 99.0%, AR), potassium hydroxide (Chengdu Chron Chem. Co., Ltd., Chengdu, China, 85.0%, AR), potassium bicarbonate (Chengdu Chron Chem. Co., Ltd., Chengdu, China, 95.0%, AR), ethanol (Shanghai Rhawn Chem. Tech. Co., Ltd., Shanghai, China, 95.0%, AR), IrO_2_ nanopowder (Suzhou Sinero Tech. Co., Ltd., Suzhou, China, 99.9%, AR), Ag nanopowder (Shanghai Aladdin Industry Co., Shanghai, China, 99.5%, 60~120 nm), carbon paper (Suzhou Sinero Tech. Co., Ltd., Suzhou, China), Nafion 117 solution (DuPont Co., Wilmington, DE, USA, 5.0 wt%), FAA-3-PK-130 membrane (Fumasep Co., Bietigheim, Germany) and Nafion 117 membrane (DuPont Co., Wilmington, DE, USA) were used without further purification.

### 2.2. Preparation of Quaternized Polybenzimidazole Membranes

**Scheme 1:**
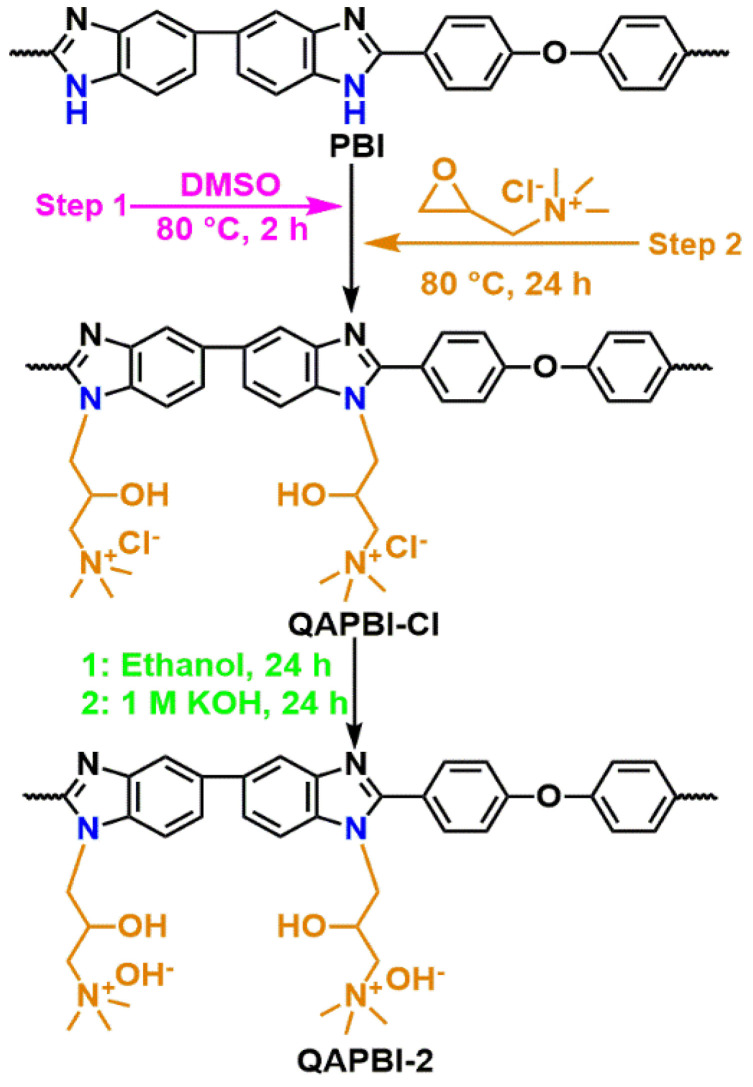
The synthetic route of a QAPBI-2 membrane.

The QAPBI membranes were prepared by adjusting the ratio of PBI and 2,3-epoxypropyltrimethylammonium chloride (EPTMA-Cl). The usage amounts of PBI and EPTMA-Cl for PBI, QAPBI-1, QAPBI-2 and QAPBI-3 membranes were calculated according to their molar ratios (see Table S1). Taking QAPBI-2 as an example, the molar ratio of PBI polymer with two N−H groups to EPTMA-Cl is 1:2. A typical preparation route of a QAPBI-2 membrane is illustrated in Scheme 1. Firstly, PBI (0.40 g) was dissolved into dimethyl sulfoxide (40.0 mL) in a flask with a magnetic stirrer at 80 °C. Subsequently, EPTMA-Cl (0.3032 g) was also added into this flask and stirred at 80 °C for 24 h to obtain a solution. Then, the above solution was poured into acetone under continuous stirring, and a solid was produced. The solid was washed repeatedly using ethanol and deionized water. Lastly, this solid was vacuum dried at 40 °C for 24 h. Thus, 0.53 g of QAPBI-2 polymer with the yield of 70.79% was successfully prepared.

Secondly, as-synthesized QAPBI-2 polymer (1.0 g) and dimethyl sulfoxide (20.0 mL) were added into a beaker with a mechanical stirrer. Whereafter, this solution was cast onto a clean glass plate and dried at 80 °C for 48 h to evaporate the solvent. The QAPBI-2 membrane was obtained and soaked into ethanol for 24 h to remove the residual solvent. Additionally, then, the QAPBI-2 membrane was placed into 1.0 mol L^−1^ KOH solution to completely replace Cl^−^ with OH^−^. The flexible QAPBI-2 membrane was obtained for further use. Compared with FAA-3-PK-130 ($2800–3000 per m^2^) and Nafion membranes ($500–700 per m^2^), the QAPBI-2 membrane ($96.85 per m^2^) has a much better cost performance for scaling up (see Table S2). Similar methods were used to prepare QAPBI-1 and QAPBI-3 membranes, whose molecular structures are presented in Figure S1. In addition, the PBI membrane was also prepared using a solution casting method.

### 2.3. Characterization of Membranes

The ATR-FTIR (Nicolet-5700 spectrometer, Thermoelectric Instrument Co., West Chester, PA, USA) and ^1^H-NMR (Bruker Advance III 600 MHz, Bruker Co., Karlsruhe, Germany) were used to investigate the chemical structures of membranes. The micro-morphologies of membranes were observed by using SEM (TM-4000, Hitachi Instrument Co., Tokyo, Japan) and AFM (SPA-300HV, Nippon Seiko Co., Tokyo, Japan). The physico-chemical properties and ECR performances of membranes were also tested. All details of above tests were described in the Supporting Information.

## 3. Results and Discussion

### 3.1. Chemical Structure Analyses

**Figure 1:**
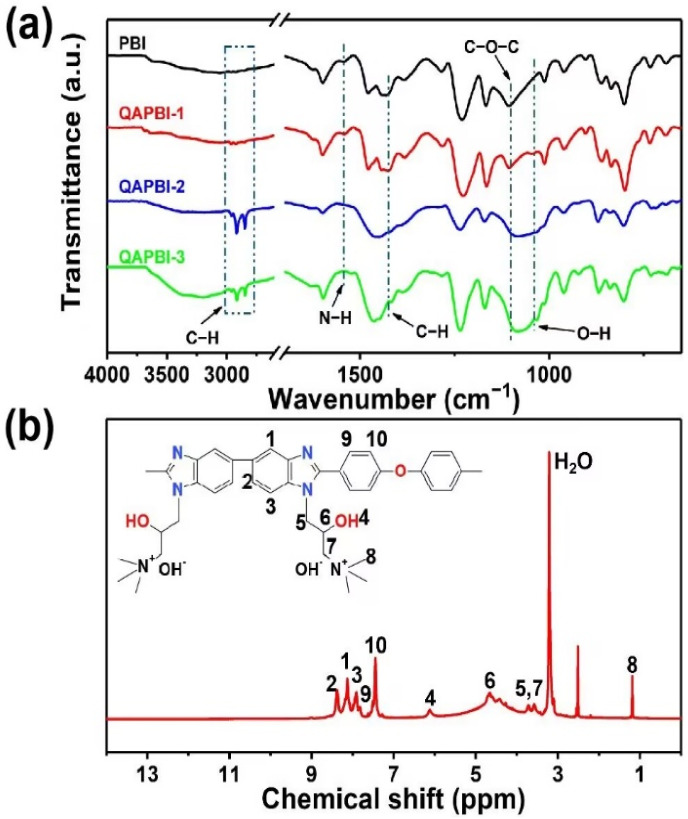
(**a**) ATR-FTIR spectra of PBI, QAPBI-1, QAPBI-2 and QAPB-3 membranes; (**b**) ^1^H-NMR spectrum of QAPBI-2 membrane.

As shown in ATR-FTIR spectra of PBI and QAPBI membranes (Figure 1a), the peak vibration of the N−H bond on imidazole groups appears at 1543 cm^−1^, which gradually weakens with the increase in grafting amount of EPTMA-Cl [21,22]. The characteristic peak at 1419 cm^−1^ is identified as the stretching vibration of C−H of the methyl and methylene groups. Additionally, two new peaks at 2852 cm^−1^ and 2920 cm^−1^ can be attributed to the symmetric and asymmetric stretching vibration of C−H, respectively [23,24]. However, the new peaks at 2852 cm^−1^ and 2920 cm^−1^ of the QAPBI-1 membrane are obviously weaker than those of the QAPBI-2 and QAPBI-3 membranes because of the low quaternization degree of the QAPBI-1 membrane. A similar result was also reported by McNair, R. et al. [25]. The stretching vibration of C−O−C appears at 1098 cm^−1^, and the peak of C−O−C on the QAPBI-3 membrane is significantly stronger than the PBI, QAPBI-1 and QAPBI-2 membranes, because excess EPTMA-Cl can react with hydroxyl groups in the side chain through a ring-opening reaction. In addition, no peak can be found at 1036 cm^−1^ for the PBI membrane; however, the O−H stretching vibration peak on the flexible akyle side chains is observed at 1036 cm^−1^ after grafting, which becomes more and more apparent with the rising grafting amount of EPTMA-Cl. Although the signature peak of O−H mixed with N−H can form a wide and strong peak at 3700 cm^−1^~2800cm^−1^ [23,24,25], this is a wavenumber range, but not a specific wavenumber. Thus, the characteristic peak of O−H at around 3600 cm^−1^ is not mentioned in this work. The ATR-FTIR results demonstrate that the EPTMA-Cl has been grafted onto imidazole groups of PBI polymer through a ring opening reaction as expected, and the QAPBI membranes have been prepared successfully.

The ^1^H-NMR spectrum of the QAPBI-2 membrane was also tested for further proof of its chemical structure (see Figure 1b). The signal around 13.0 ppm is a proton on N−H of imidazole groups, which disappears after grafting EPTMA-Cl [26]. The chemical shifts at 7.0~8.5 ppm are derived from the hydrogens of aromatic rings of the PBI backbone [27]. The QAPBI-2 membrane shows a new peak at 1.3 ppm, which is the proton of quaternary ammonium groups [28]. The double peak at 3.3 ppm is the hydrogen of methylene, indicating that the flexible alkyl side chains have been successfully grafted to the PBI backbone through a ring-opening reaction. The peak at 4.6 ppm corresponds to the proton bonded with the tertiary carbon, and the peak at 6.1 ppm is sourced from the hydroxyl on the side chain, respectively. The above results also demonstrate that the QAPBI-2 membrane has been successfully prepared using PBI and EPTMA-Cl as raw materials.

In addition, the XPS spectrum of the QAPBI- membrane was measured, and the result is shown in Figure S2. In C1’s spectra, there are two characteristic peaks at 286.3 eV and 284.5 eV, which can be attributed to C−N and C−C, respectively [29]. In N1’s spectra, three characteristic peaks appear at 401.0 eV (the nitrogen in quaternary ammonium groups), 399.3 eV (N−H) and 397.8 eV (−N=) [30]. In O1’s spectra, two characteristic peaks appear at 532.8 eV (C−O) and 531.7 eV (O−H) [31]. The XPS results further confirm that the EPTMA-Cl has been grafted onto imidazole groups of the PBI polymer.

### 3.2. Morphological Analyses

**Figure 2:**
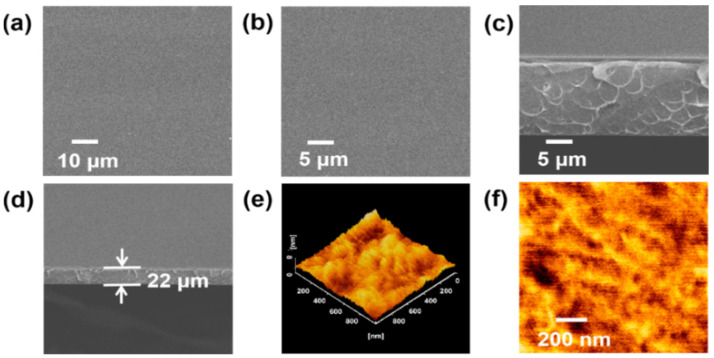
(**a**,**b**) The surface morphologies of QAPBI-2 membrane; (**c**,**d**) he cross-sectional morphologies of QAPBI-2 membrane; (**e**) The 3d-AFM image of QAPBI-2; (**f**) The 2d-AFM phase image of QAPBI-2.

The micro-morphologies of the QAPBI-2 membrane are shown in Figure 2. Notably, the surface and cross-sectional micro-morphologies of the QAPBI-2 membrane are dense and uniform without any micro-pores and micro-cracks. The thickness of the QAPBI-2 membrane is approximate 22 μm, and the fish scales are observed after freeze-fracture in liquid nitrogen due to two reasons: i) the QAPBI-2 is re-dissolved in dimethyl sulfoxide before the membrane is formed, leading to swelling between the main chains; and ii) the plastic deformation is caused by stress concentration in some weak parts of membrane. Similar fish scales after freezing were also observed by Jheng, L.C. et al. [32] and Devrim, Y. et al. [33]. The QAPBI-2 membrane with a dense structure can effectively prevent the ionic penetration [34]. Thus, the ECR performance can be improved by inhibiting the attachment of HCO_3_^−^ and CO_3_^2−^ to the cathode surface, thereby causing more CO_2_ to diffuse to the electrocatalyst in the cathode exposed.

The surface of the QAPBI-2 membrane was also observed by AFM (see Figure 2). As can be seen from Figure 2e, the surface roughness of QAPBI-2 membrane is 0.745 nm. As shown in Figure 2f, the light regions are ascribed to hydrophobic polymer backbone domains, whereas the dark regions represent the hydrophilic ionic domains [35,36]. Fortunately, the QAPBI-2 membrane (i.e., hydrophilic flexible quaternary ammonium pendants and hydrophobic aromatic backbones) has a similar micro-phase separation structure with a Nafion 117 membrane, which effectively improves the OH^−^ conduction of the QAPBI-2 membrane [37]. The result means that the ECR with the QAPBI-2 membrane could obtain excellent performance (see Section 3.6).

### 3.3. Mechanical and Thermal Properties Analyses

The mechanical properties of the PBI, QAPBI, FAA-3-PK-130 and Nafion 117 membranes are presented in Figure S3. Compared with the PBI membrane, the maximum tensile strengths of the QAPBI membranes are lower. Additionally, the maximum tensile strength of the QAPBI membrane gradually decreases with the rising grafting amount of EPTMA-Cl. This is mainly because of the disruption of hydrogen bond interactions between imidazole groups and the increase in free volume with the raised content of pendant groups [25,26]. However, the elongations at the break of QAPBI-1 and QAPBI-2 membranes are superior to those of the PBI membrane, owing to the introduction of flexible side chains [35,38]. When the grafting amount of EPTMA-Cl is further increased, the elongation at the break of the QAPBI-3 membrane is lowered due to the excessive molecular chain interactions. Compared with PBI and QAPBI membranes, the Nafion 117 membrane has a notably lower maximum tensile strength and an obviously higher elongation at break, since the Nafion 117 membrane has a special structure with a flexible aliphatic backbone and perfluorosulfonic acid side chain. Moreover, the mechanical properties of the FAA-3-PK-130 membrane are superior to QAPBI membrane; this results from the semi-crystalline properties of backbone of the FAA-3-PK-130 polymer [39,40]. In order to investigate mechanical stability, ex situ immersion experiments were performed in 0.1 mol L^−1^ KHCO_3_ and KOH solutions separately (see Figure S4). The maximum tensile strength and elongation at the break of the QAPBI-2 membrane was almost unchanged after soaking in 0.1 mol L^−1^ KHCO_3_ and KOH solutions for 3 days. The results mean that the as-prepared QAPBI-2 membrane has excellent mechanical stability for application in electrochemical CO_2_ reduction.

Thermogravimetry analysis (TGA) curves of the PBI, QAPBI and Nafion 117 membranes in nitrogen atmosphere are shown in Figure S5a. There are three distinct weight loss steps. The first weight loss from 80 to 150 °C is attributed to the loss of absorbed moisture. The second weight loss (150~300 °C) corresponds to the decomposition of quaternary ammonium groups. The third weight loss beyond 500 °C is caused by further decomposition of skeleton [41]. Moreover, the glass transition temperatures of PBI, QAPBI and Nafion 117 are around 300 °C (see Figure S5b). The electrochemical CO_2_ reduction is generally operated at room temperature, thus the as-perpared QAPBI membranes can meet the requirements well.

### 3.4. Water Uptake, Swelling Ratio and Ion Exchange Capacity Analyses

The water uptakes (*WU*s), swelling ratios (*SR*s) and ion exchange capacities (*IEC*s) of the PBI, QAPBI, FAA-3-PK-130 and Nafion 117 membranes are shown in Table 2. The *WU*s and *SR*s of QAPBI membranes are higher than those of the PBI membrane. Additionally, the *WU* and *SR* of the QAPBI membrane gradually increase with the rising grafting amount of EPTMA-Cl. This can be attributed to the raised content of quaternary ammonium groups. Compared with PBI membranes, the *IEC*s of QAPBI membranes are increased significantly, demonstrating that the QAPBI membranes with sufficient ion exchange groups can effectively transfer OH^−^. Particularly, the QAPBI-3 membrane obtains the highest *IEC* of all of the QAPBI membranes, because the hydroxyl groups can further react with EPTMA-Cl through ring opening. Such a result is also proved by the above ATR-FTIR spectra of the QAPBI membranes in Figure 1a.

### 3.5. OH^−^ Conductivity Analysis

An ideal anion exchange membrane should have high OH^−^ conductivity for ECR application. The OH^−^ conductivities of the PBI, QAPBI, FAA-3-PK-130 and Nafion 117 membranes were measured in 1.0 mol L^−1^ KOH at 20 °C (see Table 2). The OH^−^ conductivities of all QAPBI membranes (15.17~48.13 mS cm^−1^) are apparently higher than that of the PBI membrane (3.70 mS cm^−1^), which can be ascribed to two reasons: (i) The quaternary ammonium groups of QAPBI membranes are helpful for conducting OH^−^, according to the vehicle mechanism. Additonally, the hydrophilic hydroxyl and quaternary ammonium groups can effectively improve the hydration degree of the QAPBI membrane, thereby increasing the OH^−^ conductivity according to Grouthuss mechanism [17,35,42]. (ii) The flexible alkyl side chains can stretch the PBI main chain so that the QAPBI membrane obtains a larger free volume to heighten the OH^−^ conductivity [43,44]. Moreover, the trend of OH^−^ conductivity in the QAPBI membrane is a parabola with a rising grafting amount of EPTMA-Cl. This could be attributed to fact that the excess water content of the QAPBI-3 membrane leads to serious swelling, causing the concentration of quaternary ammonium groups to decrease [17,44]. Besides, the free volume of QAPBI-3 membrane could be reduced, owing to excessive molecular chain interactions. Thus, the QAPBI-2 membrane has maximum OH^−^ conductivity among all QAPBI membranes, which is obviously higher than FAA-3-PK-130 membrane. As shown in Table S3, the OH^−^ conductivity of the QAPBI-2 membrane is at a top level compared with recently reported anion exchange membranes for ECR. However, the Nafion 117 membrane has the highest OH^−^ conductivity of all membranes due to its more obvious micro-phase separation structure [37].

**Figure 3:**
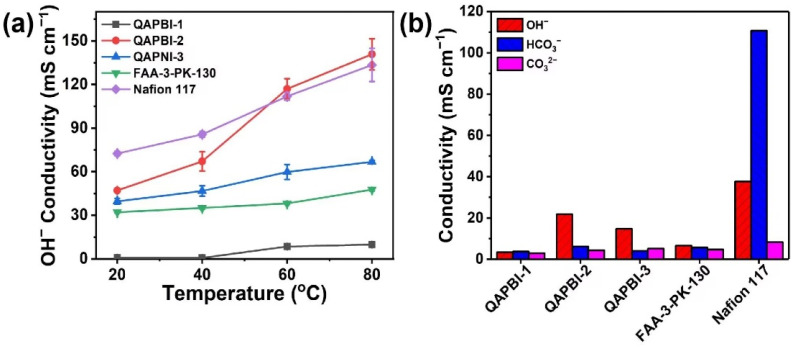
(**a**) The OH^−^ conductivities of PBI, QAPBI, FAA-3-PK-130 and Nafion 117 membranes at different temperatures. (**b**) The OH^−^, HCO_3_^−^ and CO_3_^2−^ conductivities of QAPBI, FAA-3-PK-130 and Nafion 117 membranes.

The OH^−^ conductivities of all membranes gradually increase with the rise of temperature (see Figure 3a), since the diffusion speed of OH^−^ is improved at elevated temperature [42,43]. The OH^−^ conductivities of the QAPBI-2 membrane are significantly superior to those of the FAA-3-PK-130 membrane at all temperatures. Fortunately, the OH^−^ conductivities of the QAPBI-2 membrane are higher than that of the Nafion 117 membrane when the temperature is increased above 60 °C, and the difference becomes more apparent as the temperature rises further. This could be attributed to the vibration motion of the polymer matrix and the motion of water increasing, and the system gradually reaching a relatively stable equilibrium state of thermodynamics and morphology at elevated temperature. During such an equilibrium period, randomly arranged water molecules gradually connect with each other, creating a relatively wide H-bond water network and developing a larger water channel [45]. This result means that the QAPBI-2 membrane has excellent OH^−^ conduction ability, especially at high temperatures. Moreover, an ideal anion exchange membrane ought to possess high OH^−^ conductivity and low CO_3_^2−^ and HCO_3_^−^ migration in ECR operation. Therefore, the conductivities of the QAPBI membranes are also compared in 0.1 mol L^−1^ KOH, 0.1 mol L^−1^ K_2_CO_3_ and 0.1 mol L^−1^ KHCO_3_ solutions at 20 °C separately. As shown in Figure 3b, the OH^−^ conductivity of the QAPBI-2 membrane is obviously higher than that of QAPBI-1, QAPBI-3 and FAA-3-PK-130 membranes. However, the CO_3_^2−^ and HCO_3_^−^ conductivities of the QAPBI-2 membrane are only slightly higher compared with QAPBI-1, QAPBI-3 and FAA-3-PK-130 membranes. These results prove that the QAPBI-2 membrane has an excellent ability to prevent HCO_3_^−^ and CO_3_^2−^ transfer [15]. In the aqueous solution containing CO_2_, it is inevitable to form HCO_3_^−^ and CO_3_^2−^ by neutralizing OH^−^ in the cathode. The neutralization is very fast, and almost all OH^−^ ions change to HCO_3_^−^ and/or CO_3_^2−^ within 30 min [14]. However, the QAPBI-2 membrane can selectively conduct OH^−^, which breaks the ionization balance of carbonic acid in the anolyte, causing CO_2_ to be reduced rather than causing it to form HCO_3_^−^ and/or CO_3_^2−^. Unfortunately, the HCO_3_^−^ transfer of the Nafion 117 membrane is obviously higher than that of OH^−^ conductivity, suggesting that the OH^−^ selectivity of the Nafion 117 membrane is very low. Since the alkalinity of 0.1 mol L^−1^ KHCO_3_ solution is much lower than 0.1 mol L^−1^, KOH solution, which is more beneficial for the cation-exchange membrane (Nafion 117) to transfer anions. The OH^−^ selectivity of all membranes were calculated according to Equation (S8). The OH^−^ selectivity of the QAPBI-2 membrane (67%) is higher compared with QAPBI-1 (34%), QAPBI-3 (61%), FAA-3-PK-130 (39%) and Nafion 117 (24%) membranes. Large amounts of HCO_3_^−^ and CO_3_^2−^ transmitted to anode will break the ionization balance of carbonic acid in the anolyte, making CO_2_ more inclined to react with OH^−^, thus reducing the utilization rate of CO_2_ for the Nafion 117 membrane (see Figure S6) [17]. Moreover, besides an electrochemical CO_2_ reduction, a more serious hydrogen evolution reaction tends to occur for the acidic Nafion 117 membrane in contrast with the basic QAPBI-2 membrane. In other words, the intended CO_2_ electrolysis in acids largely becomes the water electrolysis [12]. Thus, the QAPBI-2 membrane is used for ECR and compared with commercial FAA-3-PK-130 and Nafion 117 membranes.

### 3.6. ECR Performances

**Figure 4:**
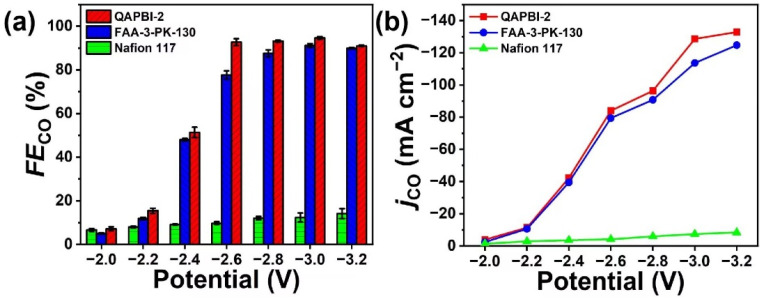
(**a**) The Faraday efficiencies of CO and (**b**) CO partial current densities of QAPBI-2, FAA-3-PK-130 and Nafion 117 membranes at various potentials in an MEA reactor.

The ECR performances of QAPBI-2, FAA-3-PK-130 and Nafion 117 membranes were evaluated under various potentials in a membrane electrode assembly (MEA) reactor and H-type cell. The ECR performances of the QAPBI-2, FAA-3-PK-130 and Nafion 117 membranes in MEA reactors are shown in Figure 4a,b. The Faraday efficiency of CO and the CO partial current density of the QAPBI-2 membrane are 95.1% and −128.4 mA cm^−2^ at −3.0 V; these are superior to FAA-3-PK-130 (Faraday efficiency of CO: 90.6% and CO partial current density: −113.6 mA cm^−2^) and Nafion 117 (Faraday efficiency of CO: 10.9%, and CO partial current density: −7.6 mA cm^−2^) membranes. This is mainly due to the higher OH^−^ selectivity of the QAPBI-2 membrane compared with FAA-3-PK-130 and Nafion 117 membranes [46]. Moreover, the Faraday efficiencies of CO of the QAPBI-2 membrane is higher than those of FAA-3-PK-130 and Nafion 117 membranes at all voltages (see Figure S7a,b) in an H-type cell. Under an optimal voltage condition of −1.5 V, the ECR performance of the QAPBI-2 membrane (Faraday efficiency of CO: 98% and CO partial current density: −33.8 mA cm^−2^) is superior to that of FAA-3-PK-130 membrane (Faraday efficiency of CO: 92% and CO partial current density: −23.8 mA cm^−2^) and Nafion 117 membrane (Faraday efficiency of CO: 96% and CO partial current density: −26.8 mA cm^−2^). It can be found from above results that the Faraday efficiency of CO of the Nafion 117 membrane is much higher in the case of an H-type cell than that in case of an MEA reactor; the reasons for this are as below. Compared with the H-type cell, the MEA reactor without an electrolyte in the cathode has a special structure, where the membrane is tightly adhered to catalyst, and the Nafion 117 membrane transports protons from anode to the catalyst surface in cathode. Additionally, the hydrogen evolution reaction occurs more easily than CO_2_ reduction in case of the MEA reactor, especially when using the acidic Nafion 117 membrane, and the Faraday efficiency of CO is greatly reduced. A similar result was also reported by Wang, G.L. et al. [47] and Ma, M. et al. [48]. Particularly, the CO partial current density of the QAPBI-2 membrane is clearly superior to FAA-3-PK-130 and Nafion 117 membranes at voltages below −0.9 V and −1.3 V. For all membranes, the CO partial current density in an MEA is higher than in an H-type cell, since CO partial current density is not limited by CO_2_ solubility in water using an MEA reactor, and the ohmic loss is minimized by decreasing the distance between two electrodes in the MEA reactor [7,12]. Compared with the reported membranes, the QAPBI-2 membrane also has an excellent Faraday efficiency of CO and CO partial current density for application in electrochemical CO_2_ reduction (see Table S4). In addition, the CO_2_ conversion rate in the MEA reactor is obviously higher than in the H-type cell for all membranes, suggesting that the MEA reactor has more excellent ECR performance (see Figure S6). More importantly, in the similar MEA, the CO_2_ conversion rate of the QAPBI-2 membrane (17.82%) is higher than that of FAA-3-PK-130 (15.77%) and Nafion 117 (1.03%) membranes. This result could be possibly because that proper content of quaternary ammonium groups not only improves the OH^−^ conductivity and selectivity, but also restrains the side reaction (CO_2_ + OH^−^ → HCO_3_^−^, CO_2_ + 2OH^−^ → CO_3_^2−^+H_2_O). Thus, the QAPBI-2 membrane can effectively enhance the CO_2_ conversion rate.

**Figure 5:**
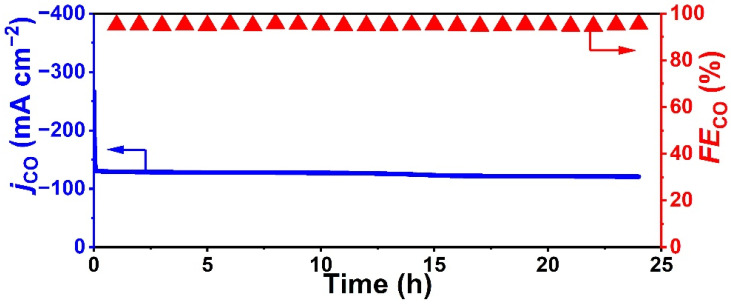
The ECR durability of QAPBI-2 membrane at −3.0 V in MEA reactor.

The durability of a membrane is an important index for ECR application. Figure 5 shows the stability of the QAPBI-2 membranes at −3.0 V in MEA reactor. The QAPBI-2 membrane exhibits excellent ECR stability in the MEA reactor (which can stably operate 24 h) and the Faraday efficiency of CO and CO partial current density maintain around 95% and −128.4 mA cm^−2^. In addition, the Faraday efficiency of the CO and CO partial current density of the QAPBI-2 membrane maintain around 98% and −33.8 mA cm^−2^ over 320 h at −1.5 V in an H-type cell (see Figure S8). Moreover, the chemical structure of the QAPBI-2 membrane was investigated to deeply evaluate its structural stability after durability testing both in an H-type cell and an MEA. As shown in Figure S9, there are no new peak appearance and shifts, meaning that the chemical structure of the QAPBI-2 membrane does not change apparently after long-term ECR usage. However, the stretching vibration peak of C−O−C is weakened after the durability test, indicating that the C−O−C is partially attacked by hydroxide.

## 4. Conclusions

In this work, three QAPBI anion exchange membranes with different quaternization degrees were successfully designed and fabricated for ECR application. Among all QAPBI membranes, the QAPBI-2 membrane has the highest OH^−^ conductivity and selectivity. Moreover, the Faraday efficiency of CO (H-type cell: 98%; MEA reactor: 95%) and the CO partial current density (H-type cell: −33.8 mA cm^−2^; MEA reactor: −128.4 mA cm^−2^) of the QAPBI-2 membrane are superior to those of commercial FAA-3-PK-130 and Nafion 117 membranes at equal voltages, respectively. Additionally, the QAPBI-2 membrane exhibits an excellent durability of 24 h and 320 h in ECR operation in an MEA reactor and an H-type cell, respectively. These results verify that the application of QAPBI-2 membrane as a potential separator in ECR will enjoy a bright future. Moreover, the polymer synthesis strategy in this work can be transferred to other polymers containing reactive −OH and −NH groups.

## Figures and Tables

**Table 1:** The reactions of electrochemical reduction of CO_2_ to CO.

	Cathode	Anode
**Reaction**	CO_2_+H_2_O+2e^−^ → CO+2OH^−^ CO_2_+2H^+^+2e^−^ → CO+H_2_O HCO_3_^−^+H^+^+2e^−^ → CO↑+2OH^−^	4OH^−^−4e^−^ → 2H_2_O+O_2_↑ 2H_2_O−4e^−^ → 4H^+^+O_2_↑
**Side reaction**	2H^+^+2e^−^ → H_2_↑ CO_2_+OH^−^ → HCO_3_^−^	

**Table 2:** Physico-chemical properties of PBI, QAPBI, FAA-3-PK-130 and Nafion 117 membranes.

Membrane	Thickness (μm)	*SR* (%)	*WU* (%)	*IEC* (mmol g^−1^)	*AR* (Ω cm^2^)	OH^−^ Conductivity (mS cm^−1^)
**PBI**	22	2.5	1.8	1.37	5.95	3.70
**QAPBI-1**	22	3.5	16.9	2.05	1.45	15.17
**QAPBI-2**	22	5.7	25.7	3.01	0.46	48.13
**QAPBI-3**	22	6.4	27.9	4.13	0.54	41.07
**FAA-3-PK-130**	130	9.6	3.4	0.93	3.89	33.42
**Nafion 117**	175	10.3	20.7	/	2.37	73.88

## Data Availability

Not applicable.

## Supplementary Materials

The following supporting information can be downloaded at: https://www.mdpi.com/article/10.3390/membranes13020166/s1, Table S1: The amounts of raw materials for preparation of membranes; Table S2: The cost evaluation of as-prepared QAPBI-2 membrane per m^2^; Table S3: Comparisons of OH^−^ conductivity of QAPBI-2 with that of other reported anion exchange membranes for ECR; Table S4: Comparisons of OH^−^ conductivity of QAPBI-2 with that of other reported anion exchange membranes for ECR; Figure S1: The structures of QAPBI-1 and QAPBI-3 membranes; Figure S2: The XPS spectrum of QAPBI-2 membrane; Figure S3: The mechanical properties of PBI, QAPBI, FAA-3-PK-130 and Nafion 117 membranes; Figure S4: The mechanical stability of QAPBI-2 membrane in (a) 0.1 mol L^−1^ KHCO_3_ and (b) 0.1 mol L^−1^ KOH for 1, 2 and 3 days; Figure S5: (a) The TGA and (b) DSC curves of PBI, QAPBI and Nafion 117 membranes; Figure S6: The CO_2_ conversion rate of QAPBI-2, FAA-3-PK-130 and Nafion 117 membranes in an H-type cell and MEA reactor; Figure S7: The Faraday efficiencies of CO and CO partial current densities of QAPBI-2, FAA-3-PK-130 and Nafion 117 membranes at various potentials in an H-type cell. Figure S8: The ECR durability of QAPBI-2 membrane at −1.5 V in an H-type cell; Figure S9: ATR-FTIR spectra of QAPBI-2 membrane before and after durability test.
