# Supplementary material for: Electrochemical Conversion of CO2 to CO Utilizing Quaternized Polybenzimidazole Anion Exchange Membrane
Source: Membranes (Basel). 2023 Jan 29;13(2):166. doi: 10.3390/membranes13020166 (PMC9961908; doi:10.3390/membranes13020166)
Supplement: Supplementary file 1 [file membranes-13-00166-s001.zip › membranes-2145624-supplementary.pdf]

Supporting Information

Electrochemical Conversion of CO_2 to CO Utilizing Quaternized Polybenzimidazole Anion Exchange Membrane

Jingfeng Li [1], Zeyu Cao [1], Bo Zhang [1], Xinai Zhang [1], Jinchao Li [1,2,*], Yaping Zhang [2,*], Hao Duan [3]

[1] State Key Laboratory of Environment–friendly Energy Materials, Engineering Research Center of Biomass Materials (Ministry of Education), and School of Materials Chemistry, Southwest University of Science and Technology, Mianyang, 621010, P.R. China

[2] School of Chemical Engineering, Sichuan University, Chengdu, 610065, P.R. China

[3] Sichuan Langsheng New Energy Technology Co. Ltd, Suining, 629200, P.R. China

* Correspondence: lijinchao@swust.edu.cn; zhangyaping@swust.edu.cn; Tel.: +86-816-6089372

This file includes:

Experimental section

Tables S1 to S4

Figures S1 to S9

1. Experimental

1.1 The pre-treatment process of Nafion 117 membrane

Nafion 117 membrane was pretreated according to the following method. Firstly, Nafion 117 membrane was pretreated in 3.0 wt% H_2O_2 solution at 70 °C for 0.5 h and then taken out and washed with deionized water. Afterwards, Nafion 117 membrane was immersed in 0.05 mol L^{-1} H_2SO_4 solution at 70 °C for 0.5 h and then taken out and washed with deionized water. Finally, the pretreated Nafion 117 membrane was stored in deionized water for further use.

1.2 ATR-FTIR, ^{1}H-NMR, XPS, SEM and AFM measurements

The scanning range and resolution of attenuated total reflectance Fourier transform infrared (ATR-FTIR) spectra (Nicolet-5700 spectrometer, Thermoelectric Instrument Co., USA) are 400-4000 cm^{-1} and 4 cm^{-1}, respectively. The deuterium dimethyl sulfoxide was used as a solvent in ^{1}H-NMR measurement (Bruker Advance III 600 MHz, Germany). X-ray photoelectron spectroscopy (XPS, Thermo Scientific ESCALAB Xi+, USA) is also used to analyze the binding energy of as-prepared membrane. Besides, the surface and cross-sectional morphologies were observed by using scanning electron microscope (SEM) (TM-4000, Hitachi Instrument Co., Japan), and the cross-section of membrane was obtained using the freeze-fracture technique in liquid N_2. The atomic force microscopy (AFM) (SPA-300HV, Nippon Seiko Co., Japan) was used to investigate surface morphology and phase structure of membrane.

1.3 Mechanical and thermal properties tests

The mechanical properties of dry membrane were measured using an electromechanical universal testing machine (QJ-210A, Shanghai Instrument Co., China) at room temperature with a tensile speed of 10 mm min^{-1}. The maximum tensile strength (*TS*) and elongation at break (*EB*) were calculated according to Eqs. (S1-S2):

$$TS = \frac{F}{d \times w} \tag{S1}$$

$$EB = \frac{L'}{L} \tag{S2}$$

where *F* represents the maximum stress (N) at break, *d* and *w* indicate the thickness (μm) and width (cm) of membrane, respectively. *L*' and *L* are the absolute elongation (mm) at break and the initial length (mm) of membrane, individually.

The thermogravimetry (TG) and differential scanning calorimetry (DSC) of membranes were measured by synchronous thermal analyzer (Netzsch STA449F3, Germany) at a heating rate of 10 °C min^{-1} under the nitrogen atmosphere and the temperature range from room temperature to 800 °C.

1.4 Water uptake, swelling ratio and ion exchange capacity tests

The water uptake (*WU*) and swelling ratio (*SR*) of membrane were tested according to the following procedures. Firstly, the membrane (1 cm × 4 cm) was dried at 40 °C for 24 h in order to remove moisture totally, and the weight

and thickness were measured. Then, the membrane was soaked in deionized water at room temperature for 24 h. After the membrane was taken out from the deionized water, the excess water on the membrane surface was removed by using a piece of filter paper. Finally, the weight and thickness of the membrane were immediately measured again. The *WU* and *SR* of membrane were calculated according to Eqs. (S3-S4):

$$WU = \frac{M_w - M_d}{M_d} \times 100\% \qquad \text{(S3)}$$

$$SR = \frac{T_w - T_d}{T_d} \times 100\% \qquad \text{(S4)}$$

where M_d/T_d is the weight (g)/thickness (μm) of dried membrane, and M_w/T_w is the weight (g)/thickness (μm) of wetted membrane.

The ion exchange capacity (*IEC*) of membrane was measured by traditional acid-base titration. The membrane was dried at 40 °C for 24 h, and then its weight was measured. The dry membrane was immersed into 0.1 mol L^{-1} HCl solution for 24 h to complete ion exchange. Lastly, the soaking solution was titrated with 1.0 mol L^{-1} NaOH solution. The *IEC* of membrane was calculated according to Eq. (S5):

$$IEC = \frac{V_{HCl}C_{HCl} - V_{NaOH}C_{NaOH}}{M_d} \qquad \text{(S5)}$$

where C_{HCl}/C_{NaOH} and V_{HCl}/V_{NaOH} are the concentration (mol L^{-1}) and volume (mL) of HCl/NaOH solution individually.

1.5 Area resistance, OH^- conductivity and OH^- selectivity tests

The area resistance (*AR*) of membrane was measured by constant current

electrochemical impedance spectroscopy. The membrane was immersed into 1.0 mol L^{-1} KOH solution at room temperature for 24 h. Then, the H-type conductivity cells were filled with 1.0 mol L^{-1} KOH solution at 20 °C, 40 °C, 60 °C and 80 °C respectively. The impedance values of H-type conductivity cells with and without the membrane were measured, respectively. The *AR* and OH^- conductivity (σ) of membrane were calculated according to Eqs. (S6-S7):

$$AR = (R_1 - R_0) \times A' \tag{S6}$$

$$\sigma = \frac{T}{AR} \tag{S7}$$

where A' is the effective area (1.76 cm^2) of membrane, R_1 and R_0 represent the impedance values (Ω) of the H-type conductivity cells with and without the membrane respectively, T is the thickness of membrane (μm).

In addition, the OH^-, HCO_3^- and CO_3^{2-} conductivities of membrane in 0.1 mol L^{-1} KOH, 0.1 mol L^{-1} $KHCO_3$ and 0.1 mol L^{-1} K_2CO_3 solutions were also measured at room temperature. The OH^- selectivity of membrane was calculated according to Eq. (S8):

$$x_{OH^-} = \frac{\sigma_{OH^-}}{\sigma_{OH^-} + \sigma_{HCO_3^-} + \sigma_{CO_3^{2-}}} \times 100\% \tag{S8}$$

where σ_{OH^-}, $\sigma_{HCO_3^-}$ and $\sigma_{CO_3^{2-}}$ are OH^-, HCO_3^- and CO_3^{2-} conductivities (mS cm^{-1}) of membrane, respectively.

1.6 Electrochemical CO_2 reduction tests in H-type cell and membrane electrode assembly (MEA) reactor

The electrochemical CO_2 reduction (ECR) of membrane was evaluated by using an H-type cell. The Ag foil electrode was immersed in a CO_2-saturated 0.1 mol L^{-1} $KHCO_3$ electrolyte in the H-type cell, where Pt and Ag/AgCl were used as a counter electrode and a reference electrode, respectively. The membrane was used as a separator, which was immersed into 1.0 mol L^{-1} KOH solution at room temperature for 24 h before the ECR measurement. The H-type cell was filled with 0.1 mol L^{-1} $KHCO_3$ solution. The Ag_2CO_3 layer was synthesized on the Ag foil electrode by applying an anodic potential of 2.6 V for 3 min. Then, the Ag_2CO_3 layer was reduced to metallic Ag in the initial period of ECR. CO_2 with a flow rate of 20 mL min^{-1} was supplied to the cathode. The chronoamperometry test was conducted by applying the constant voltage. The composition of ECR products were analyzed by a gas chromatography (GC 9790Plus, Fuli instrument, Co., Ltd, China). Besides, the durability of membrane was also investigated by using continuous electrolysis with a constant voltage until the CO partial current density occurs decay.

MEA was fabricated by using a catalyst-coated diffusion layer method. The cathode catalyst ink was prepared by mixing 5.0 mg of Ag nanopowder with 250 μL of deionized water, 750 μL of isopropyl alcohol and 50 μL of 5.0 wt% Nafion 117 ionomer solution, followed by ultra-sonicating for 30 min. The anode catalyst ink was prepared by mixing 7.0 mg of IrO_2 nanopowder with 250 μL of deionized water, 750 μL of isopropyl alcohol and 60 μL of 5.0

wt% Nafion 117 ionomer solution, followed by ultra-sonicating for 30 min. The cathode and anode electrodes were prepared by spraying the metal catalyst ink on the carbon paper. The MEA was prepared by sandwiching the electrodes and membrane between gas flow channels. CO_2 with a flow rate of 20 mL min^{-1} and 0.1 mol L^{-1} $KHCO_3$ was supplied to the cathode and anode by peristaltic pump, respectively. The effective area of membrane is 4.0 cm^2. The chronoamperometry test was conducted by applying the constant voltage.

The CO_2 conversion rate (CR_{CO_2}) reflects the conversion degree of CO_2 to CO. The Faraday efficiency of CO (FE_{CO}), CO partial current density (j_{CO}, mA cm^{-2}) and CR_{CO_2} were calculated according to Eqs. (S9-S11):

$$FE_{CO} = \frac{\alpha n F}{Q} \times 100\% \quad (S9)$$

$$j_{CO} = FE_{CO} \times j_{total} \quad (S10)$$

$$CR_{CO_2} = \frac{n}{n_{CO_2}} \times 100\% \quad (S11)$$

where α is the number of transferred electrons of ECR (α=2), n is the number of moles of generated CO (mol), F is Faraday constant (96485 C mol^{-1}), Q is the actual coulomb (C) consumed in electrolysis process, j_{total} is total current density of cathode (mA cm^{-2}), n_{CO_2} is the number of moles (mol) of CO_2.

2. Supplementary Tables

Table S1 The amounts of raw materials for preparation of PBI and QAPBI membranes.

Membrane	PBI	EPTMA-Cl
**PBI**	0.40 g (1.0 mmol)	/
**QAPBI-1**	0.40 g (1.0 mmol)	0.1516 g (1.0 mmol)
**QAPBI-2**	0.40 g (1.0 mmol)	0.3032 g (2.0 mmol)
**QAPBI-3**	0.40 g (1.0 mmol)	0.4548 g (3.0 mmol)

Table S2 The cost evaluation of as-prepared QAPBI-2 membrane per m^2.

Component	Unit price (RMB)	Consumption	Cost (RMB)
**Polybenzimidazole**	22.00 g^{-1}	16.67 g	366.74
**2,3-epoxypropyltrimethylammonium chloride**	0.88 g^{-1}	13.00 g	11.44
**Dimethyl sulfoxide**	0.04 mL^{-1}	1.00 L	40.00
**Acetone**	0.03 mL^{-1}	3.00 L	90.00
**Ethanol**	0.01 mL^{-1}	2.00 L	20.00
**Deionized water**	0.95 L^{-1}	2.00 L	1.90
**Electric energy**	0.52 kW h^{-1}	240 kW	124.80
**Total**	**RMB 654.88≈$ 96.85**		

Table S3 Comparisons of OH^- conductivity of QAPBI-2 with that of other reported anion exchange membranes for ECR.

Membrane	Temperature	Electrolyte	OH^- Conductivity	Reference
**QPPEEK-PEG 20**	20 °C	1.0 mol L^{-1} KOH	Around 28 mS cm^{-1}	[S1]
**QPBI-DA**	30 °C	deionized water	23.1 mS cm^{-1}	[S2]
**PES-8$C_{0.15}$-$QA_{0.85}$**	30 °C	1.0 mol L^{-1} KOH	Around 28 mS cm^{-1}	[S3]
**A_8SB-QAPPO**	30 °C	1.0 mol L^{-1} KOH	40.8 mS cm^{-1}	[S4]
**PGG–GH**	RT	deionized water	24 mS cm^{-1}	[S5]
**[PVDBIm]30[OH]**	30 °C	deionized water	1.66 mS cm^{-1}	[S6]
**aQAPS**	20 °C	deionized water	Around 35 mS cm^{-1}	[S7]
**DQ-PPO-17-OH**	20 °C	deionized water	Around 40 mS cm^{-1}	[S8]
**Tec-PBI-30**	30 °C	deionized water	Around 30 mS cm^{-1}	[S9]
**QAPBI-2**	**20 °C**	**1.0 mol L^{-1} KOH**	**48.13 mS cm^{-1}**	**This work**

Table S4 Comparisons of the ECR performance of QAPBI-2 with that of other reported membranes.

Membrane	Temperature	Reduction Product	Device	FE_{CO}/durability	Current density	Reference
**FAA-3-PK-130 (Cu/CNT)**	40 °C	CO	MEA	Around 96%/1000 s	Around −30-mA cm^{-2}	[S10]
**Nafion 115 (Cu/CNT)**	40 °C	CO	MEA	Around 6%/1000 s	Around −90 mA cm^{-2}	[S10]
**QAPPT (Au/C, H_2O)**	60 °C	CO	MEA	96%/100 h	−100 mA cm^{-2}	[S11]
**PVDF PM (Ag, 0.5 mol L^{-1} $KHCO_3$)**	RT	CO	MEA	80%/100 h	Around −150-mA cm^{-2}	[S12]
**Ralex® BM (Ag, 1 mol L^{-1} KOH)**	RT	CO	MEA	65%/55 h	Around −25 mA cm^{-2}	[S13]
**Ag-BPM-Pt (Ag, 1 mol L^{-1} NaOH)**	RT	CO	MEA	90%/10 h	>−10 mA cm^{-2}	[S14]
**FAA-3-50 (Cu, 1 mol L^{-1} KOH)**	RT	CO	MEA	Around 30%/--	Around −90 mA cm^{-2}	[S15]
**QAPBI-2 (Ag, 0.1 mol L^{-1} $KHCO_3$)**	**20 °C**	**CO**	**MEA**	**95%/24 h**	**−128.4 mAcm^{-2}**	**This work**

3. Supplementary Figures

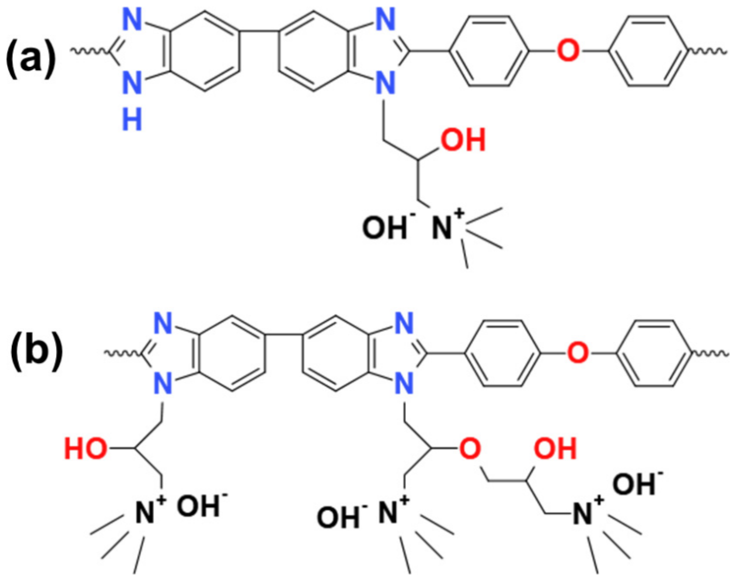

Figure S1. The structures of (a) QAPBI-1 and (b) QAPBI-3 membranes.

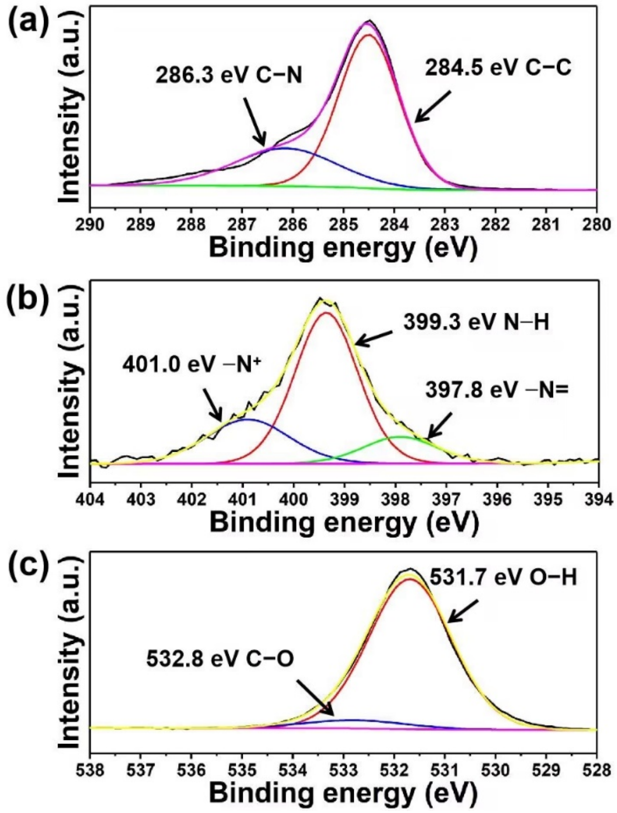

Figure S2. The XPS spectrum of QAPBI-2 membrane.

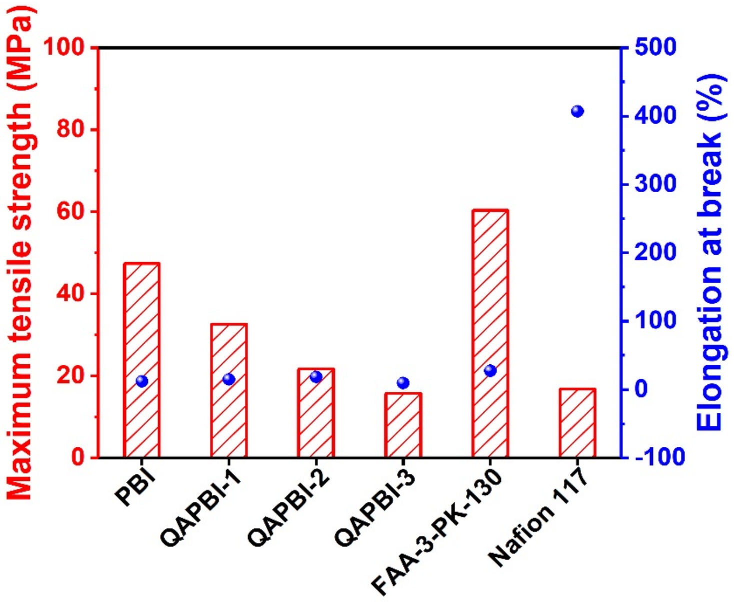

Figure S3. The mechanical properties of PBI, QAPBI, FAA-3-PK-130 and Nafion 117 membranes.

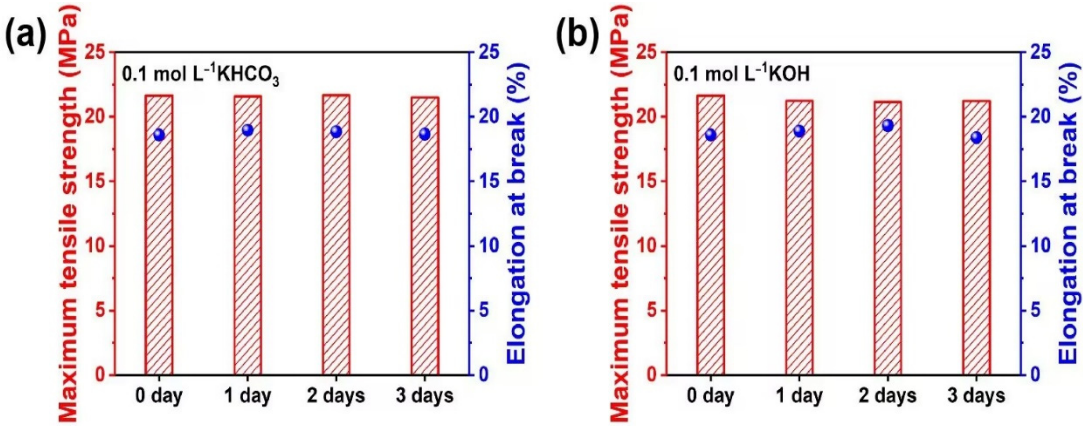

Figure S4. The mechanical stability of QAPBI-2 membrane in (a) 0.1 mol L^{-1} $KHCO_3$ and (b) 0.1 mol L^{-1} KOH for 1, 2 and 3 days.

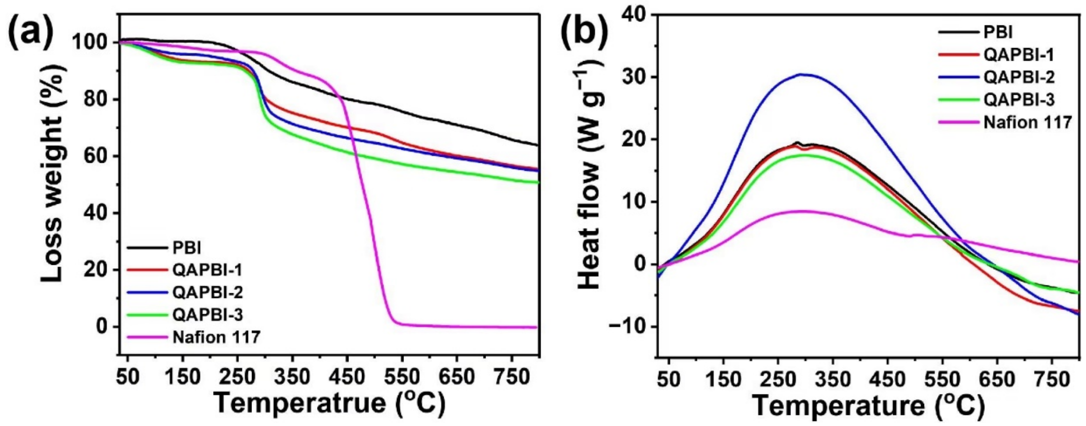

Figure S5. (a) The TGA and (b) DSC curves of PBI, QAPBI and Nafion 117 membranes.

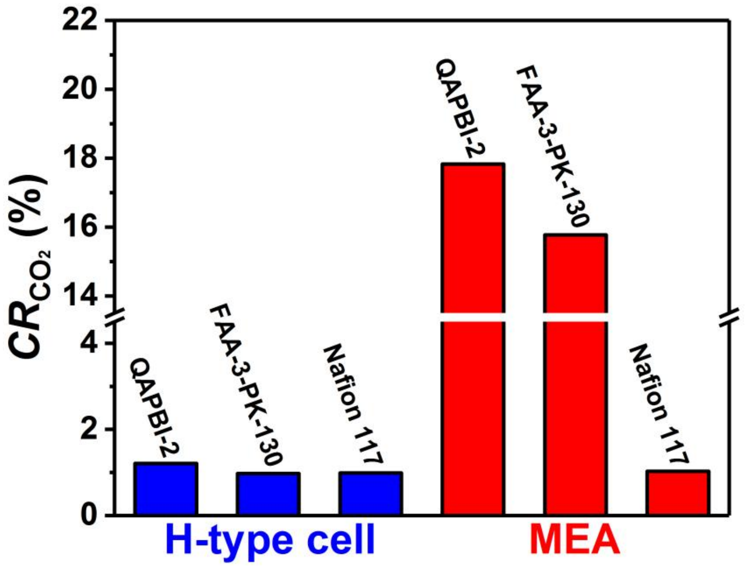

Figure S6. The CO_2 conversion rate of QAPBI-2, FAA-3-PK-130 and Nafion 117 membranes in an H-type cell and MEA reactor.

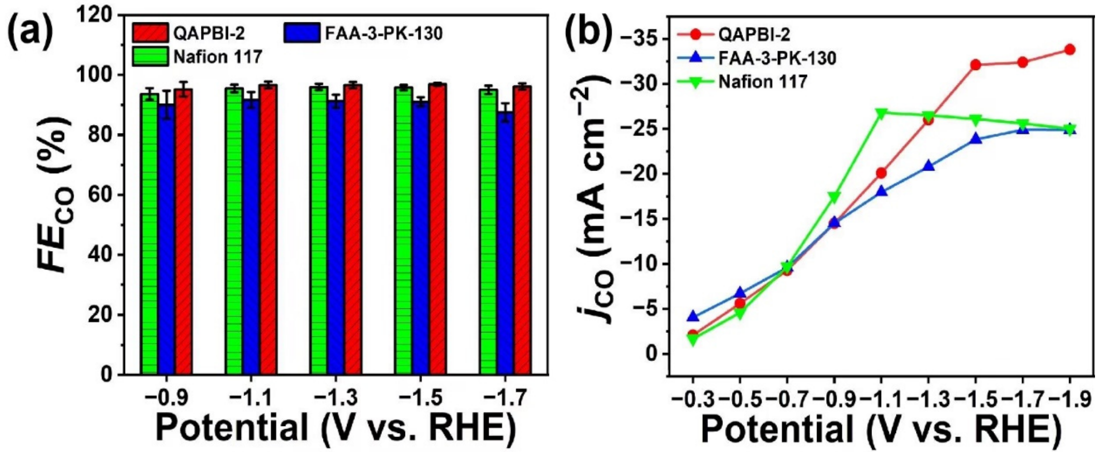

Figure S7. (a) The Faraday efficiencies of CO and (b) CO partial current densities of QAPBI-2, FAA-3-PK-130 and Nafion 117 membranes at various potentials in an H-type cell.

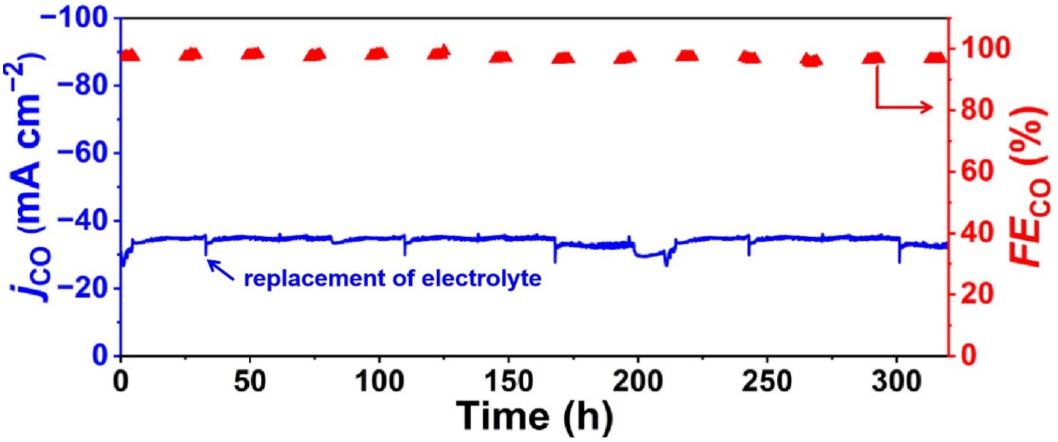

Figure S8. The ECR durability of QAPBI-2 membrane at −1.5 V in an H-type cell.

During the electrolysis process in H-type cell, the fluctuation of CO partial current density is resulted from the consumption/production of water in the catholyte/anolyte, leading to the imbalance of concentrations of $KHCO_3$ solution. Thereby, the electrolytes of anode and cathode need to be replaced in ECR durability test.

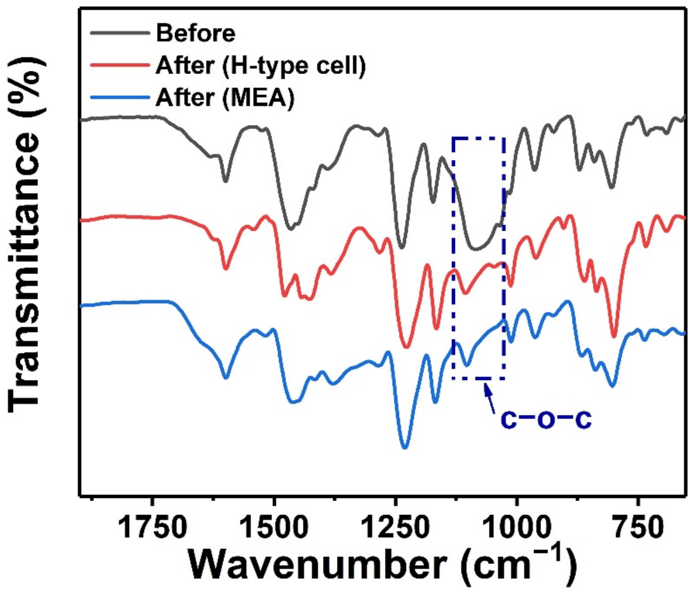

Figure S9. ATR-FTIR spectra of QAPBI-2 membrane before and after durability test.

4. Supplementary References

[S1]Kumari, M.; Douglin, J.C.; Dekel, D.R. Crosslinked quaternary phosphonium-functionalized poly(ether ether ketone) polymer-based anion-exchange membranes. J. Membr. Sci. **2021**, 626, 119167.

[S2]Wang, X.Z.; Chen, W.T.; Yan, X.M.; Li, T.T.; Wu, X.M.; Zhang, Y.; Zhang, F.; Pang, B.; He, G.H. Pre-removal of polybenzimidazole anion to improve flexibility of grafted quaternized side chains for high performance anion exchange membranes. J. Power Sources **2020**, 451, 227813.

[S3]Lin, C.X.; Liu, X.; Yang, Q.; Wu, H.Y.; Liu, F.H.; Zhang, Q.G.; Zhu, S.M.; Liu, Q.L. Hydrophobic side chains to enhance hydroxide conductivity and physicochemical stabilities of side-chain-type polymer AEMs. J. Membr. Sci. **2019**, 585, 90-98.

[S4]Han, J.J.; Gong, S.Q.; Peng, Z.S.; Cheng, X.Q.; Li, Y.H.; Peng, H.Q.; Zhu, Y.C.; Ren, Z.D.; Xiao, L.; Zhuang, L. Comb-shaped anion exchange membranes: Hydrophobic side chains grafted onto backbones or linked to cations. J. Membr. Sci. **2021**, 626, 119096.

[S5]Wang, M.; Preston, N.; Xu, N.N.; Wei, Y.N.; Liu, Y.Y.; Qiao, J.L. Promoter effects of functional groups of hydroxide-conductive membranes on advanced CO2 electroreduction to formate. ACS Sustain. Chem. Eng. **2019**, 11, 6881-6889.

[S6]Si, Z.H.; Qiu, L.H.; Dong, H.L.; Gu, F.L.; Liu, Y.Y.; Yan, F. Effects of substituents and substitution positions on alkaline stability of imidazolium cations and their corresponding anion exchange membranes. ACS Appl. Mater. Interfaces **2014**, 6, 4346-4355.

[S7]Pan, J.; Chen, C.; Zhuang, L.; Lu, J.T. Designing advanced alkaline polymer electrolytes for fuel cell applications. Acc. Chem. Res. **2012**, 45, 473-481.

[S8]Wu, J.R.; Wei, X.T.; Jiang, H.; Zhu, Y.Q. Synthesis and properties of anion conductive polymers containing dual quaternary ammonium groups without beta-hydrogen via CuAAC click chemistry. Polymer **2021**, 228, 123920.

[S9]Wang, X.Z.; Chen, W.T.; Li, T.T.; Yan, X.M.; Zhang, Y.; Zhang, F.; Wu, X.M.; Pang, B.; Li, J.N.; He, G.H. Ultra-thin quaternized polybenzimidazole anion exchange membranes with throughout OH^- conducive highway networks for high-performance fuel cells. J. Mater. Chem. A. **2021**, 12, 7522-7530.

[S10]Wang, G.L.; Pan, J.; Jiang, S.P.; Yang, H. Gas phase electrochemical conversion of humidified CO_2 to CO and H_2 on proton-exchange and alkaline anion-exchange membrane fuel cell reactors. J. CO_2 Util. **2018**, 23, 152-158.

[S11]Yin, Z.L.; Peng, H.Q.; Wei, X.; Zhou, H.; Gong, J.; Huai, M.M.; Xiao, L.; Wang, G.W.; Lu, J.T.; Zhuang, L. An alkaline polymer electrolyte CO_2 electrolyzer operated with pure water. Energy Environ. Sci. **2019**, 12, 2455-2462.

[S12]Lee, W.H.; Kim, K.; Lim, C.; Ko, Y.J.; Hwang, Y.J.; Min, B.Y.; Lee, U.; Oh, H.S. New strategies for economically feasible CO_2 electroreduction using a porous membrane in zero-gap configuration. J. Mater. Chem. A **2021**, 9, 16169-16177.

[S13]Blommaert, M.A.; Sharifian, R.; Shah, N.U.; Nesbitt, N.T.; Smith, W.A.; Vermaas, D.A. Orientation of a bipolar membrane determines the dominant ion and carbonic species transport in membrane electrode assemblies for CO_2 reduction. J. Mater. Chem. A, **2021**, 9, 11179-11186.

[S14]Vermaas, D.A.; Smith, W.A. Synergistic electrochemical CO_2 reduction and water oxidation with a bipolar membrane. ACS Energy Lett. **2016**, 6, 1143-1148.

[S15]Zhang, J.; Luo, W.; Züttel, A. Crossover of liquid products from electrochemical CO_2 reduction through gas diffusion electrode and anion exchange membrane. J. Catal. **2020**, 385, 140-145.
